# The Impact of an Ex Vivo Pediatric Extracorporeal Membrane Oxygenation Circuit on Sequestration of Antimicrobials

**DOI:** 10.1097/CCE.0000000000001338

**Published:** 2025-10-30

**Authors:** Michele L. Cree, Mohd Hafiz Abdul-Aziz, Emma Haisz, Steven C. Wallis, Hayoung Won, Chandra D. Sumi, Dusan Marjanovic, Jenny L. Ordóñez, Luregn J. Schlapbach, Jason A. Roberts

**Affiliations:** 1 UQ Centre for Clinical Research, Faculty of Medicine, The University of Queensland, Brisbane, QLD, Australia.; 2 Pharmacy Department, Queensland Children’s Hospital, Brisbane, QLD, Australia.; 3 Pediatric Intensive Care Unit, Queensland Children’s Hospital, Brisbane, QLD, Australia.; 4 Centre for Children’s Health Research, The University of Queensland, Brisbane, QLD, Australia.; 5 Department of Intensive Care and Neonatology, and Children`s Research Center, University Children’s Hospital Zurich, University of Zurich, Zurich, Switzerland.; 6 Department of Intensive Care Medicine, Royal Brisbane & Women’s Hospital, Brisbane, QLD, Australia.; 7 Herston Infectious Diseases Institute (HeIDI), Level 8, UQ Centre for Clinical Research Royal Brisbane and Women’s Hospital, Brisbane, QLD, Australia.; 8 Division of Anesthesia Critical Care and Emergency and Pain Medicine, UR UM 103, University of Montpellier, Nimes University Hospital, Nimes, France.; 9 Pharmacy Department, Royal Brisbane and Women’s Hospital, Brisbane, QLD, Australia.

**Keywords:** antimicrobials, ex vivo, extracorporeal membrane oxygenation, pediatric, sequestration

## Abstract

**OBJECTIVES:**

To determine if common antimicrobials (*n* = 11) are sequestered or degraded during a pediatric extracorporeal membrane oxygenation (ECMO) simulation.

**DESIGN:**

An ex vivo model of a closed ECMO circuit was established to simulate the treatment of a 3 kg infant. A control was used to quantify spontaneous antimicrobial degradation.

**SETTING:**

University research laboratory.

**PARTICIPANTS:**

None.

**MAIN OUTCOMES AND MEASURES:**

The ECMO circuit was primed and maintained at physiologic pH and temperature for 7 hours. After baseline sampling, the antimicrobials were administered as a single bolus into the circuit. Eight plasma samples were taken from the controls and ECMO circuits over 7 hours. Antimicrobial concentrations were measured using validated high-performance liquid chromatography–tandem mass spectrometry methodology. The antimicrobial recovery was compared with baseline. Each simulation was performed in triplicate to assess simulation variability.

**RESULTS:**

The recovery mean (%) in ECMO at 7 hours for ampicillin 78%, cefotaxime 92%, flucloxacillin 72%, meropenem 81%, micafungin 72%, piperacillin 84%, and voriconazole 42% was significantly different from the baseline (*p* < 0.05). The recovery in the control at 7 hours for ampicillin 83%, cefotaxime 76%, flucloxacillin 90%, gentamicin 85%, meropenem 76%, piperacillin 92%, and tazobactam 93% was also significantly different from the baseline (*p* < 0.05). A significant relationship was identified in the ECMO model between the antimicrobial recovery (%) and the log partition coefficient (log *p*) of the studied antimicrobials (*R*^2^ = 0.52; *p* = 0.01). No significant relationship was identified between the protein binding and antimicrobial recovery (*R*^2^ = 0.23; *p* = 0.13).

**CONCLUSIONS AND RELEVANCE:**

The lipophilicity of an antimicrobial is a predictor of antimicrobial recovery in ECMO. Concentrations were significantly reduced at 7 hours for greater than 60% of the study antimicrobials in the ECMO models. Clinical studies are required for children receiving ECMO to determine if the current dosing regimens for antimicrobials provide therapeutic concentrations.

**KEY POINTS**

**Question**: Are common antimicrobial (antibiotic and antifungal) concentrations reduced during a pediatric extracorporeal membrane oxygenation (ECMO) treatment?

**Findings**: Reduced antimicrobial concentrations were identified in greater than 60% of the study antimicrobials during the pediatric ECMO treatment. The physiochemical property of lipophilicity was identified as a predictor of antimicrobial recovery in the pediatric ECMO model.

Extracorporeal membrane oxygenation (ECMO) provides life-saving organ support for critically ill children with acute respiratory and/or cardiac failure (1, 2). The ECMO circuit comprises of multiple components (oxygenator, pump, heat exchanger, hemofilter, and tubing) where drugs can be sequestered (3, 4). The blood and crystalloid fluids used to prime and maintain ECMO circuit flows may also lead to drug hemodilution (1, 2). Together, drug sequestration and hemodilution during ECMO can alter the pharmacokinetics of vulnerable drugs, potentially impacting their pharmacological activity (2, 5). Optimal dosing during ECMO is more challenging for certain drugs. For example, sedatives can be titrated to the desired clinical effect, whereas there are no reliable clinical markers to guide antibiotic or antifungal (antimicrobials) dosing beyond drug concentrations. Unfortunately, therapeutic drug monitoring is not available for many antimicrobials, making it difficult to determine if the antimicrobial dosing regimen is effective. Achieving optimal antimicrobial concentrations in critically ill children receiving ECMO is crucial to prevent treatment failure and mitigate the risk of antimicrobial resistance (2, 5).

Physicochemical properties of antimicrobials, such as protein binding and lipophilicity, can influence their disposition within an ECMO circuit (4, 5). Lipophilic antimicrobials (log *p* > 2) or highly protein bound antimicrobials (> 70%) have been shown to be prone to sequestration in ECMO circuits (4–6). Hydrophilic or low-to-moderate plasma protein binding antimicrobials, such as ceftolozane and tazobactam (7), ceftaroline (8), and vancomycin (9, 10), have demonstrated reduced concentrations in ex vivo pediatric ECMO studies. In pediatric patients receiving ECMO, hemodilution has also been reported to alter the volume of distribution for gentamicin and fluconazole (11), which may result in subtherapeutic concentrations. Both sequestration and hemodilution may alter the distribution of antimicrobials in critically ill children receiving ECMO, potentially resulting in subtherapeutic concentrations. Furthermore, when continuous antimicrobial infusions are used there is a risk of spontaneous antimicrobial degradation especially for meropenem (12, 13), leading to subtherapeutic antimicrobial concentrations. Despite these concerns, the same antimicrobial dosing regimen is currently used for critically ill children, regardless of ECMO support. However, there is a lack of data to confirm if this approach is appropriate.

Determining if antimicrobials are sequestered in critically ill children receiving ECMO remains a challenge. Ex vivo studies provide a valuable understanding of the interaction between an antimicrobial and the extracorporeal circuits (14). This study is the largest study to date to describe antimicrobial recovery in an ex vivo pediatric ECMO model. The aim of this study was to investigate whether common antimicrobials concentrations are reduced as a result of sequestration onto a pediatric ECMO circuit.

## METHODS

### Institutional Review Board

This study did not involve human or animal subjects, institutional review board (IRB) approval was obtained for the blood products by the Children’s Health Queensland IRB and The University of Queensland IRB (STUDY66639, “Ex Vivo Characterisation of Pediatric Extracorporeal Therapy [Extracorporeal Membrane Oxygenations (ECMO)] and Extracorporeal Continuous Renal Replacement Therapy [CRRT] on Antimicrobials Concentrations),” approved October 28, 2020) was conducted in accordance with ethical standards of The University of Queensland IRB and the Helsinki Declaration of 1975.

### Experiments in an Ex Vivo ECMO Model

Each ECMO circuit consisted of a Quadrox ID pediatric membrane oxygenator (Maquet, Cardiopulmonary GmBH, Rastatt, Germany), a rotaflow centrifugal pump (Getinge, Gothenburg, Sweden), a heat exchanger, ST20 hemofilter (surface area of 0.2 m^2^), and 1/8-inch polyvinyl chloride (PVC) tubing. The blood products (< 5 d old) were obtained through a material supply agreement with the Australian Red Cross Lifeblood (Kelvin Grove, QLD, Australia). The closed loop circuit was primed according to the local clinical protocol with 100 mL of albumin 20% (CSL Behring, Broadmeadows, VIC, Australia), 20 mmol of sodium bicarbonate 8.4% (Phebra, Sydney, NSW, Australia), 2 mmol of calcium chloride (Phebra, Australia), 1 U of red cell concentrate, and 200 U of heparin (Pfizer, Sydney, NSW, Australia). A reservoir bag containing 120 mL of whole blood and 5000 U of unfractionated heparin was connected to maintain the circuit pressure. The final ECMO model consisted of a volume of 500 mL, a treatment time of 7 hours with a flow rate of 134 mL/min to simulate a 3 kg infant. The temperature, hematocrit and pH values were maintained at physiologic conditions (pH of 6.8–7.5). Blood gases, electrolytes, and activated clotting time were measured using an i-STAT portable blood analyzer (Osborne Park, WA, Australia). A schematic diagram of the closed loop ECMO simulation and sampling sites are shown in **eFigure 1** (https://links.lww.com/CCX/B566). The ECMO simulation was performed in triplicate.

Once the ECMO circuit was stable, the study antimicrobials (ampicillin 2.5 mg, cefotaxime 2.5 mg, flucloxacillin 2.5 mg, fluconazole 1.5 mg, gentamicin 2 mg, meropenem 2.5 mg, micafungin 2.5 mg, piperacillin 4 mg, tazobactam 0.5 mg, vancomycin 2.5 mg, and voriconazole 1 mg) were prepared as an antimicrobial stock solution before being added into the circuit as a combined single bolus. Antimicrobial doses were calculated to achieve the expected plasma concentrations in a critically ill infant. Visual assessments indicated no interactions among the antimicrobials. Physicochemical properties of antimicrobials are summarized in **Table 1** (11, 15–23).

**TABLE 1.** Physiochemical Properties of Study Antimicrobials

Antimicrobial	Protein Binding (%)		Log *p*	Predicted Blood Concentration in Extracorporeal Membrane Oxygenation Circuit (mg/L)	Source
	Neonates	Child			
Ampicillin	10 (12)	15–30 (12)	1.4 (13)	4.9	Mylan, Carol Park, QLD, Australia
Cefotaxime	NR	35–40 (14–15)	–1.4 (13–15)	4.9	Hospira, Mulgrave, VIC, Australia
Flucloxacillin	75 (16)	95–97 (16)	2.6 (17)	4.9	Juno Pharmaceuticals, South Yarra, VIC, Australia
Fluconazole	NR	11–12 (15)	0.5 (15)	3	Sigma, Berrinba, QLD, Australia
Gentamicin	< 10 (15–18)	30 (13–15–19)	–3.1 (5–15)	3.9	Pfizer, Sydney, NSW, Australia
Meropenem	NR	2 (13–15)	–0.7 (5–15)	4.9	Fresenius Kabi, Mount Kuring-gai, NSW, Australia
Micafungin	90 (15)	96 (15)	–1.5 (15)	4.9	Astellas Pharma, Macquarie Park, NSW, Australia
Piperacillin	30 (15–20)	16–48 (15–20)	0.5 (13–15)	7.9	AFT Pharmaceuticals, North Ryde, NSW, Australia
Tazobactam	NR	30 (15–20)	–0.9 (13–15)	1	AFT Pharmaceuticals, North Ryde, NSW, Australia
Vancomycin	10–20 (15–21)	10–55 (13–15)	–1.4 (5–15)	4.9	Sandoz, North Sydney, NSW, Australia
Voriconazole	NR	60 (15)	2.6 (15)	2	Pfizer, Sydney, NSW, Australia

NR = not reported in the published literature.

The controls were prepared in triplicate alongside the ECMO model in PVC jars, each containing 50 mL of human whole blood and 2500 U of unfractionated heparin (Pfizer, Australia) for anticoagulation. A single bolus of study antimicrobials (ampicillin 1.25 mg, cefotaxime 1.25 mg, flucloxacillin 1.25 mg, fluconazole 0.75 mg, gentamicin 1 mg, meropenem 1.25 mg, micafungin 1.25 mg, piperacillin 2 mg, tazobactam 0.25 mg, vancomycin 1.25 mg, and voriconazole 0.5 mg) was added, resulting in a final volume of 55 mL. The antimicrobial doses used in the controls were selected to produce a similar blood concentration to those in the pseudo-patient (reservoir). The jars were placed in an incubator at 37°C and agitated continuously to ensure uniform distribution of the antimicrobials.

### Sample Collection

Pre- and post-oxygenator samples (3 mL) were collected at baseline (0 hr) and at approximately 0.5, 1.5, 2, 4, 6, and 7 hours after antimicrobial administration in the ECMO simulation, along with control samples (3 mL) over the same 7-hour period. The samples were immediately centrifuged (3000*g* × 5 min), and the plasma was separated and stored in cryovials at –80°C until bioanalysis.

### Measurement of Antimicrobials Samples in Plasma

The total plasma antimicrobial concentrations were measured using a validated high-performance liquid chromatography–tandem mass spectrometry method on a Nexera ultra high performance liquid chromatograph connected to an 8030+ triple quadrupole mass spectrometer (Shimadzu, Kyoto, Japan). All antimicrobial samples were assayed alongside a calibration standards and quality control samples and met the acceptance criteria (precision range of 3.7–9.1% with an accuracy range of 2.3–10.4%).

### Antimicrobial Recovery

The antimicrobial recovery in ECMO circuits and controls at each sampling time point was calculated using the following equation:


$$\text{Antimicrobial}\;\text{recovery}\left(\%\right)=\left(\left(\frac{\text{Ci}-\text{Ct}}{\text{Ci}}\right)\times 100\right)$$

Ci = concentration at baseline or time zero,

Ct = concentration at time “t.”

### Statistical Analysis

A linear regression analysis was performed to compare the mean antimicrobial recovery over time between ECMO circuits and controls. The percentage recovery of antimicrobial was modeled as a function of time and group differences (ECMO vs. control) were assessed by evaluating the slopes of the regression lines. Additionally, a linear regression model was used to evaluate the association between physicochemical characteristics (protein binding and log *p*) and the percentage of antimicrobial recovery at 7 hours. Statistical analyses were performed using GraphPad Prism, Version 10 (GraphPad Software, La Jolla, CA), with a two-sided *p* value of less than 0.05 considered statistically significant in all analyses.

## RESULTS

An ex vivo Quadrox ID pediatric ECMO circuit was maintained under physiologic conditions (pH, 6.8–7.5) for the 7-hour simulation without complications. The hematocrit concentrations ranged from 0.19 to 0.44 during the ECMO simulations. For each antimicrobial 66 samples were analyzed. The treatment time was 6.42 ± 0.58 hours. Overall, no statistical differences were identified in the simulations and controls, except in one of the simulations the plasma concentrations of cefotaxime, piperacillin, and tazobactam were undetectable, and plasma concentrations for flucloxacillin were 50% lower than expected from an experimental error.

The reported volumes (mean ± sd) in the study at baseline for the reservoirs (includes the blood-crystalloid mixture and antimicrobials) of 132 ± 3 mL, the ECMO circuit volumes of 380 ± 13 mL, and the total volumes (reservoir and ECMO) of 513 ± 10 mL, reporting a difference of 26% ± 1%. A measured concentration difference of 19.5% ± 2.4% was reported between the ECMO circuits and controls for the study antimicrobials.

**Figure 1.**
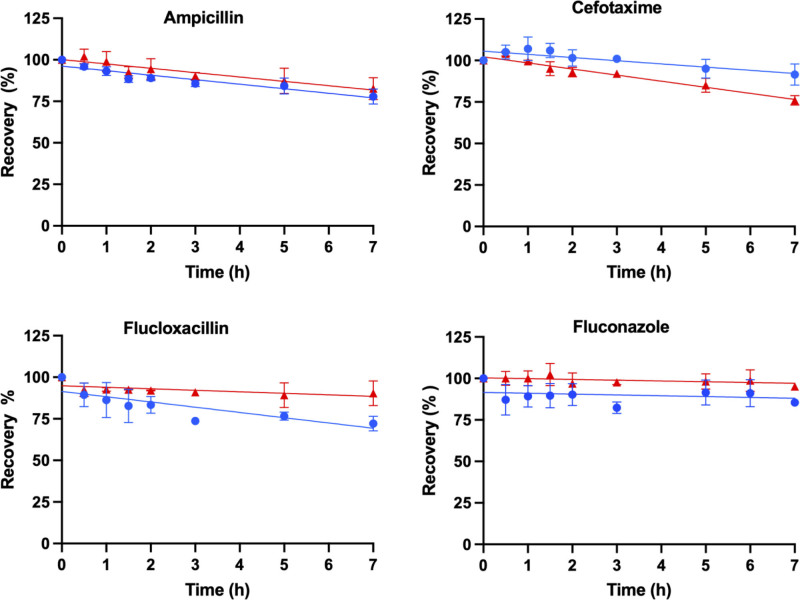
Percentage of recovery in the pediatric extracorporeal membrane oxygenation (ECMO) simulation over time (hr) for ampicillin, cefotaxime, flucloxacillin, and fluconazole, with the mean (sd) for ECMO (*blue*) and control (*red*) and a line of best fit.

**Figure 2.**
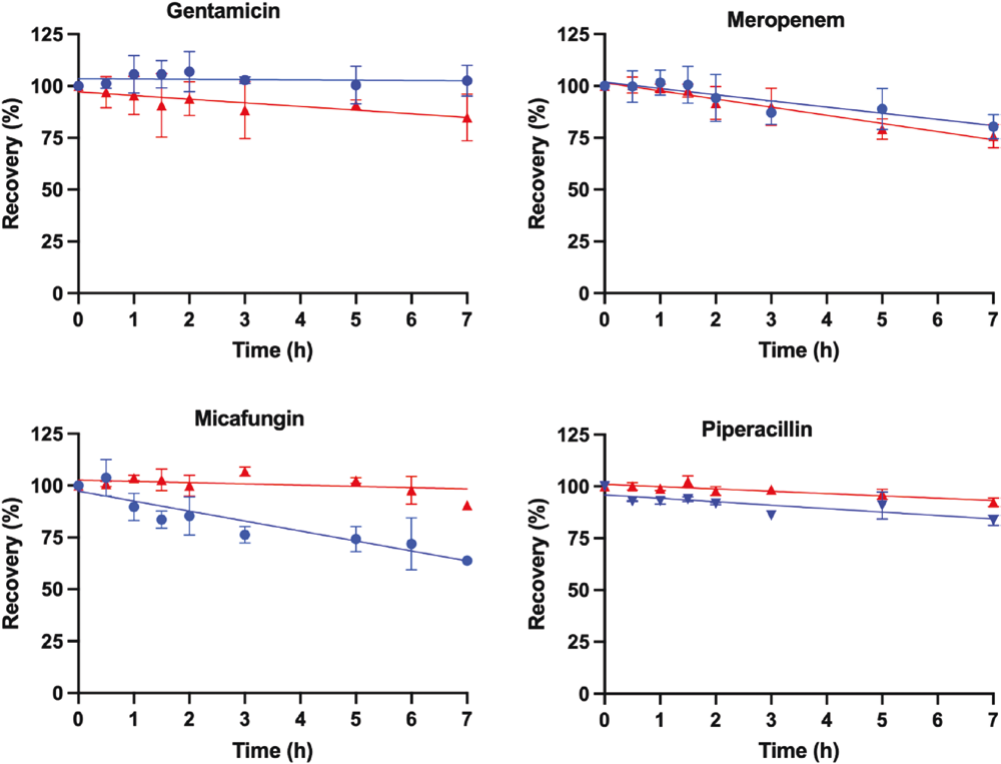
Percentage of recovery in the pediatric extracorporeal membrane oxygenation (ECMO) simulation over time (hr) for gentamicin, meropenem, micafungin, and piperacillin, with the mean (sd) for ECMO (*blue*) and control (*red*) and a line of best fit.

**Figure 3.**
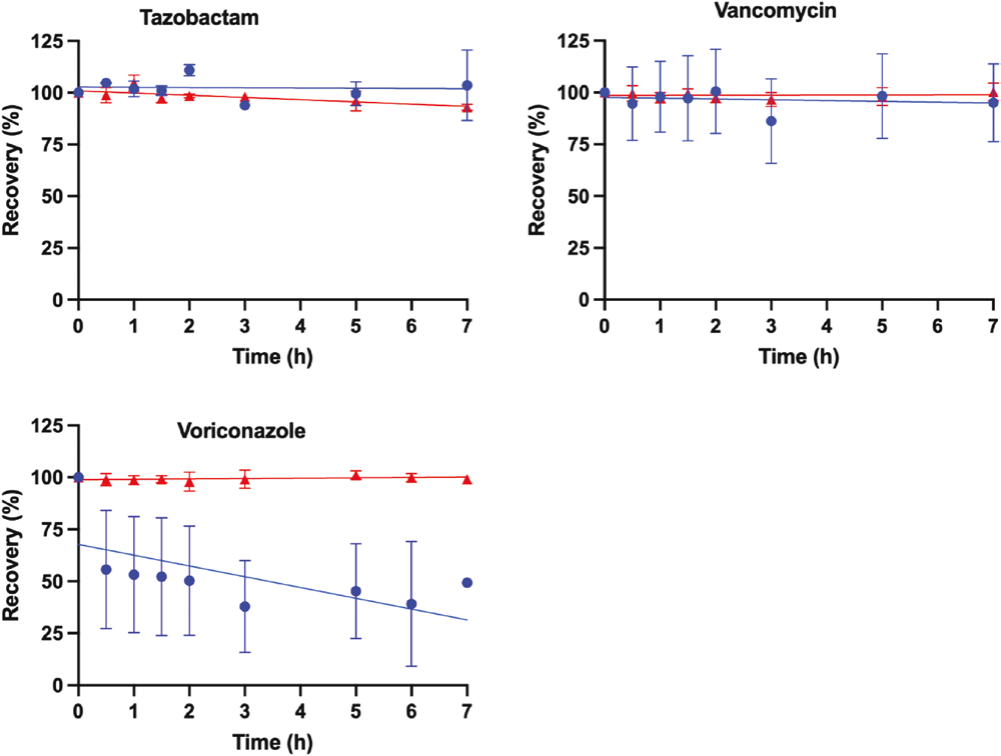
Percentage of recovery in the pediatric extracorporeal membrane oxygenation (ECMO) simulation over time (hr) for tazobactam, vancomycin and voriconazole, with the mean (sd) for ECMO (*blue*) and control (*red*) and a line of best fit.

The percentage recovery of each antimicrobial (mean ± sd) was modeled as a function of time (hr) for the samples (ECMO and control) shown in **Figures 1–3**. The mean (± sd) antimicrobial recovery at 7 hours relative to the baseline in ECMO controls and circuit are presented in **Table 2**. Mean recoveries in ECMO circuits were significantly lower than baseline concentrations for ampicillin (*p* < 0.001), cefotaxime (*p* = 0.018), flucloxacillin (*p* = 0.001), meropenem (*p* < 0.001), micafungin (*p* < 0.001), piperacillin (*p* < 0.001), and voriconazole (*p* = 0.047). In the controls, significant reductions from baseline were observed for ampicillin (*p* < 0.001), cefotaxime (*p* < 0.001), gentamicin (*p* = 0.03), meropenem (*p* < 0.001), piperacillin (*p* < 0.001), and tazobactam (*p* = 0.008). Mean recoveries between the ECMO circuits and controls were significantly different for cefotaxime (*p* = 0.016), flucloxacillin (*p* = 0.004), gentamicin (*p* = 0.045), micafungin (*p* < 0.001), and voriconazole (*p* = 0.036). No significant variability in pH was reported affecting the antimicrobial disposition in the ECMO simulations.

**TABLE 2.** Antimicrobial Recovery in Extracorporeal Membrane Oxygenation Circuits and Controls Relative to Baseline at 7 Hours

Antimicrobials	Extracorporeal Membrane Oxygenation, Mean ± sd, %	*p*	Control, Mean ± sd, %	*p*	Overall Lines Different *p*
Ampicillin	78 ± 5	**< 0.001**	83 ± 7	**< 0.001**	0.70
Cefotaxime	92 ± 5	**0.018**	76 ± 2	**< 0.001**	**0.016**
Flucloxacillin	72 ± 4	**0.01**	90 ± 10	0.062	**0.004**
Fluconazole	91 ± 9	0.457	99 ± 7	0.265	0.96
Gentamicin	103 ± 8	0.813	85 ± 13	**0.03**	**0.045**
Meropenem	81 ± 6	**< 0.001**	76 ± 7	**< 0.001**	0.29
Micafungin	72 ± 14	**< 0.001**	98 ± 7	0.145	**< 0.001**
Piperacillin	84 ± 2	**< 0.001**	92 ± 2	**< 0.001**	0.11
Tazobactam	104 ± 12	0.884	93 ± 1	**0.008**	0.26
Vancomycin	95 ± 21	0.792	100 ± 5	0.873	0.80
Voriconazole	42 ± 25	**0.047**	100 ± 2	0.459	**0.036**

Bold entries indicate statistically significant (*p* < 0.05).

**Figure 4.**
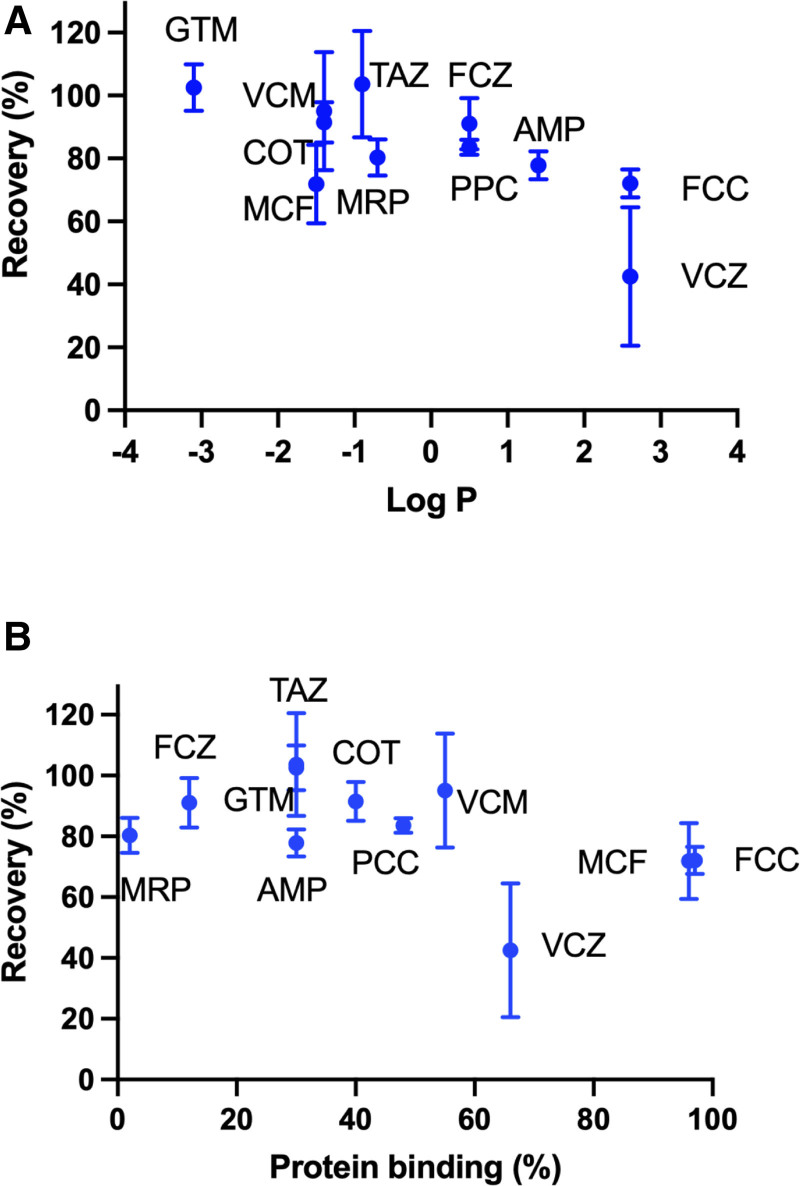
Recovery of antimicrobials in extracorporeal membrane oxygenation at 7 hr. **A**, Lipophilicity expressed as log partition coefficient (log *p*) values. **B**, Protein binding expressed as percentage. For each drug, the mean recovery is indicated by a *circle* and the upper and lower 95% CIs are indicated by *crossbars*. AMP = ampicillin, COT = cefotaxime, FCC = flucloxacillin, FCZ = fluconazole, GTM = gentamicin, MCF = micafungin, MRP = meropenem, PCC = piperacillin, TAZ = tazobactam, VCM = vancomycin, VCZ = voriconazole.

The relationship between lipophilicity (log *p*) and protein binding (%) with the antimicrobial recovery (%) from the ECMO circuits at 7 hours are presented in **Figure 4**. A significant relationship was identified between log *p* (*R*^2^ = 0.52; *p* = 0.01) and antimicrobial recovery in ECMO. No significant relationship was identified between protein binding (*R*^2^ = 0.23; *p* = 0.13) and antimicrobial recovery in ECMO.

## DISCUSSION

This ex vivo study is the largest study to date to describe the impact of a pediatric ECMO circuit on antimicrobials concentrations over 7 hours. The recovery of 11 antimicrobials was modeled over time in the pediatric ECMO model, identifying reduced concentrations in greater than 60% of the antimicrobials at 7 hours, and a dependency on the physiochemical property of an antimicrobial and the recovery (Figs. 1–3). Lipophilicity was identified as a determinant of antimicrobial recovery in ECMO (Fig. 4). This finding is of clinical significance for pediatric patients with severe infection receiving ECMO with therapeutic drug monitoring not currently available for all antimicrobials. Our findings were compared with published ex vivo studies, and although they may differ this is predominantly due to these studies being performed in adult ECMO configurations (**eTable 1**, https://links.lww.com/CCX/B566) (4, 9, 10, 24–32). This ex vivo study provides insights into the impact of pediatric ECMO on antimicrobial concentrations.

Sequestration during ECMO has been reported to be dependent on the drug physiochemical properties, lipophilicity (log *p* > 2), and protein binding (> 70%) (5, 6). However, inconsistencies exist in the published ex vivo adult ECMO studies, with one study identifying a significant relationship between the recovery and the physiochemical properties of an antimicrobial (5), whereas other studies not did identify a significant relationship (28, 33). In our ex vivo study, the majority of antimicrobials have a log *p* value of less than 2 and have low to moderate protein binding (Table 1) suggesting a low potential for sequestration in ECMO, except for flucloxacillin, micafungin, and voriconazole. In our ECMO study, a strong association was identified between the lipophilicity (log *p* > 2) and the recovery (Fig. 4). The recovery for flucloxacillin (71%), micafungin (72%), and voriconazole (42%) in the ECMO model demonstrates sequestration (Figs. 1–3) related to the drug physiochemical properties. An ex vivo adult ECMO study investigating oxacillin, an antibiotic with similar physiochemical properties (log *p*, 2.4, and protein binding [95%]) to flucloxacillin, identified 46% recovery at 48 hours (34). Similarly, an ex vivo pediatric and adult study in ECMO investigating voriconazole identified a recovery range of 33–73% for voriconazole at 24 hours (30, 32). The recovery for micafungin in our study is similar to results reported in an ex vivo adult ECMO study identifying 67% recovery at 24 hours (28). The authors in these published ex vivo studies (28, 30, 32, 34), concluded that the reduced concentrations was a result of sequestration onto the ECMO circuitry. We were unable to identify a correlation between protein binding and recovery in our ECMO model, and a longer observation period may identify a relationship. The configuration of the ECMO circulation (silicone membrane oxygenator with a centrifugal pump or a poly methylpentene (PMP) membrane oxygenator with a centrifugal or diagonal pumps) may account for the variability in the antimicrobial recovery in the published studies (eTable 1, https://links.lww.com/CCX/B566) (4, 9, 10, 24–32). Further research is required in the clinical setting for infants receiving ECMO to identify if sequestration reduces antimicrobial concentrations, and dose adjustment is required.

Spontaneous antimicrobial degradation has been reported for amoxicillin, cefepime, meropenem, and piperacillin in human plasma samples over 24 hours (35). Our ex vivo study, identified reduced concentrations for ampicillin, cefotaxime, flucloxacillin, gentamicin, meropenem, piperacillin, and tazobactam in the control at 7 hours (Table 2 and Figs. 1–3). These reduced concentrations may indicate spontaneous degradation, or delayed bioanalysis (> 6 mo storage period), or delayed processing of the samples or temperature storage conditions are not maintained (36). Further research is required in clinical setting for infants receiving ECMO to identify if spontaneous degradation reduces antimicrobial concentrations.

In this ex vivo study, a volume difference of 26% was identified between the reservoir (pseudo-patient) and the total volume in ECMO model, and a concentration difference of 20% was reported between the measured concentrations for ECMO and control, which may represent hemodilution during ECMO. The blood and crystalloid volume required to prime or maintain flows in an ECMO treatment for a neonate or an infant can be twice the blood volume of an infant, this additional blood volume can alter the volume of distribution of hydrophilic antimicrobials (2, 37) and lead to subtherapeutic concentrations. Furthermore, the ongoing need for blood products and crystalloids to maintain flows during an ECMO treatment may further alter the distribution of the antimicrobials (1). Hemodilution may not be clinically significant is an adult receiving ECMO, as the patient to ECMO blood volume ratio is not a factor. However, further research is required in the clinical setting for infants receiving ECMO to identify if hemodilution reduces antimicrobial concentrations.

Our study identified significantly reduced concentrations in ECMO for ampicillin, cefotaxime, flucloxacillin, meropenem, micafungin, piperacillin, and voriconazole (*p* < 0.05) at 7 hours. This finding indicates a need to review the antimicrobial dosing regimens for critically ill children receiving ECMO. Previous ex vivo studies report predominantly on the recovery of a single antimicrobial in either an adult ECMO circuit or pediatric ECMO configurations with silicone oxygenators (eTable 1, https://links.lww.com/CCX/B566) (4, 9, 10, 24–32). Furthermore, there are no studies to compare that describe gentamicin’s recovery in an ex vivo pediatric ECMO model and only one study that describes ampicillin, cefotaxime, and flucloxacillin recovery in an adult ex vivo ECMO model at 24 hours (27). Pediatric ECMO circuits are not the same as adult ECMO circuits whereby the pump and oxygenator are not integrated (38) (eFig. 1, https://links.lww.com/CCX/B566), and little is known if this difference may alter the total surface area of the circuit. Different extracorporeal membrane oxygenators (silicone vs. PMP) (9, 24, 28) and pumps (centrifugal vs. roller) (25–29) have demonstrated reduced antimicrobial concentrations in ECMO. A study using a silicone oxygenator identified reduced concentrations for meropenem (28) and vancomycin (9), with the centrifugal pumps reporting highly variable concentrations for meropenem (9, 24, 28). In addition, the age of an ECMO circuit has been shown to impact the degree of antimicrobial saturation (9), and the use of new circuits in the ex vivo study only reflects first dose, with future clinical studies required to determine the impact of repeated antimicrobial doses on the ECMO circuit. Our study strived to emulate a pediatric ECMO clinical configuration, with similar tubing volume, a blood-crystalloid fluid prime and maintaining a physiologic pH throughout the simulation; however, the reduced antimicrobial concentrations reported does not necessarily equate to clinical practice, with critical illness and organ dysfunction playing a role in antimicrobial distribution and clearance. Clinical studies are required to determine if the current dosing regimens for antimicrobials are therapeutic in children receiving ECMO to minimize treatment failure or the development of resistant pathogens.

There are several limitations in the ex vivo study. The use of donated blood does not reflect real-life infants, clinical situations, and the metabolism of the anticoagulant used in the blood in the circuit (to prevent blocking the circuit) is not possible, leading to ongoing hemolysis that may affect the measurement of antimicrobial concentrations (14). The variability in hematocrit and pH in the simulations may explain the difference in the predicted plasma accuracy for the antimicrobials. For future studies, defining the antimicrobial concentrations in whole blood (rather than plasma) may remove the variability identified with the hematocrit. One of the ECMO simulations reported no detectable plasma concentrations for cefotaxime, piperacillin, tazobactam, and 50% lower plasma concentrations for flucloxacillin from an experimental error in preparation of the antimicrobial stock solution, and as such this simulation was excluded in the statistical analysis for these antimicrobials. In the controls, only the temperature was monitored, and it is possible that differences in recovery may be a consequence of changes in pH or light exposure. Although, the pre-oxygenator samples identified reduced concentrations for all of the antimicrobials these results were not significantly different from the post-oxygenator samples for the same time point indicating that sequestration may be a cumulative and difficult to detect during a short time period or that the antimicrobials may be sequestered to other components in the ECMO circuit. Previous studies have reported on the antimicrobial recovery over 24–48 hours (eTable 1, https://links.lww.com/CCX/B566) (4, 9, 10, 24–32), in our study, the observational time period was 7 hours, with the majority of the antimicrobials dosed every 6–8 hours this time period was deemed sufficient. Our study described the antimicrobial recovery in a pediatric ECMO circuit over 7 hours, it did not describe the clinical patient setting or the effect of the individual components on the antimicrobial concentrations, this was outside the scope of this study. In addition, this study did not describe the impact of competitive drug binding or drug saturation during ECMO.

## CONCLUSIONS

This ex vivo study has identified a significant relationship between log *p* and the antimicrobial recovery in a pediatric ECMO model, it was unable to identify a significant relationship for protein binding. In greater than 60% of the antimicrobials, the concentrations in the ECMO circuit were reduced from baseline and may result in subtherapeutic concentrations and treatment failure. Our findings differ from previous ex vivo studies conducted in adult circuits or in different ECMO configurations (including the oxygenators, pumps and tubing, and the composition of the priming fluids). Future clinical studies are required to explore the interaction between the critically ill child and an ECMO circuit to determine if sequestration, degradation, or hemodilution result in subtherapeutic antimicrobial concentrations and treatment failure.

## Supplementary Material


