# Supplementary material for: The Impact of an Ex Vivo Pediatric Extracorporeal Membrane Oxygenation Circuit on Sequestration of Antimicrobials
Source: Crit Care Explor. 2025 Oct 30;7(11):e1338. doi: 10.1097/CCE.0000000000001338 (PMC12577806; doi:10.1097/CCE.0000000000001338)
Supplement: Supplementary file 1 [file cc9-7-e1338-s001.pdf]

Supplemental Digital content

1. **eFigure 1. (a) A schematic of the pediatric *ex-vivo* ECMO circuit model** with the sampling points (pre- and post- oxygenator) highlighted in purple. FdO2, fraction of delivered oxygen. **(b)** A schematic of the adult *ex-vivo* ECMO circuit model
2. **eTable 1.** Recovery of antimicrobials in *ex-vivo* ECMO studies over time.

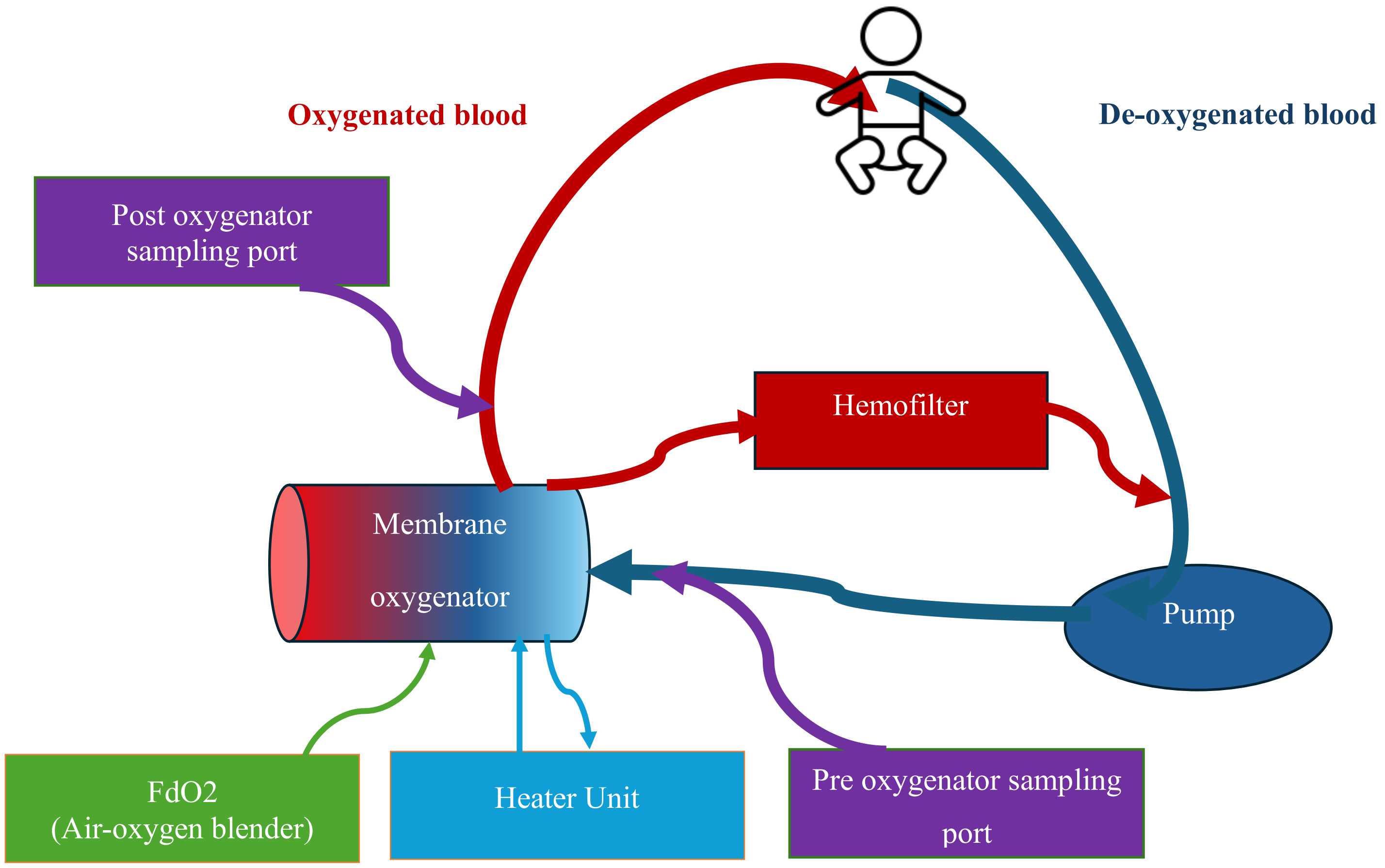

(a)

eFigure 1. (a) A schematic of the pediatric ECMO circuit *ex-vivo* model with the sampling points (pre- and post- oxygenator) highlighted in purple. FdO2; fraction of delivered oxygen. **(b)** A schematic of the adult ECMO circuit *ex-vivo* model.

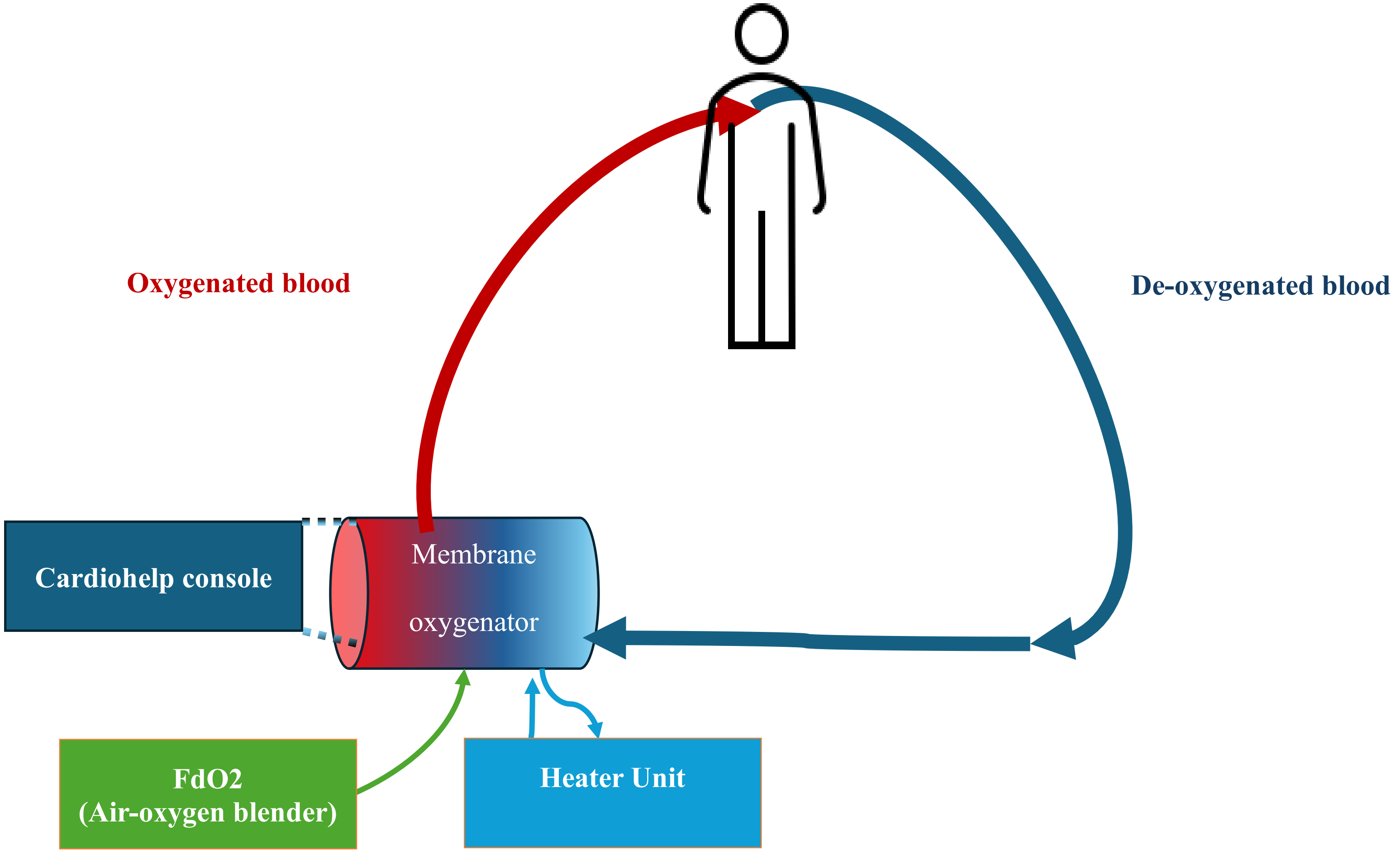

(b)

eFigure 1. (a) A schematic of the pediatric ECMO circuit *ex-vivo* model with the sampling points (pre- and post- oxygenator) highlighted in purple. FdO2; fraction of delivered oxygen. **(b)** A schematic of the adult ECMO circuit *ex-vivo* model.

3. **eTable 1.** Recovery of antimicrobials in *ex-vivo* ECMO studies over time.

**Study**	**Duration of laboratory (hour)**	**Type of oxygenator**	**Type of pump**	**Percent remaining (%)**									
				**Ampicillin**	**Cefotaxime**	**Flucloxacillin**	**Fluconazole**	**Gentamicin**	**Meropenem**	**Micafungin**	**Piperacillin**	**Vancomycin**	**Voriconazole**
**Study**	**7**	**PMP MO**	**Centrifugal**	**78**	**92**	**72**	**91**	**100**	**81**	**72**	**84**	**95**	**42**
Mehta[a], 2007 [22]	24	Silicone MO	NR	85	NR	NR	NR	NR	NR	NR	NR	91[c]	29
Wildschut[a], 2010 [23]	3	Silicone MO	Roller	NR	NR	NR	NR	NR	*83**	NR	NR	*68**	NR
Wildschut[a], 2010 [23]	3	PMP MO	Centrifugal	NR	NR	NR	NR	NR	89^	NR	NR	67^	NR
Shekar[b], 2012 [24]	7	PMP MO	Centrifugal	NR	NR	NR	NR	NR	65	NR	NR	100	NR
Lemaitre[b], 2015 [25]	48	PMP MO	Centrifugal	NR	NR	NR	NR	NR	NR	NR	NR	100	NR
Shekar[b], 2015 [4]	24	PMP MO	Centrifugal	NR	NR	NR	91	NR	20	NR	NR	91	NR
Leven[b],2017 [26]	24	PMP MO	Centrifugal	84	46	62	NR	NR	NR	NR	79	NR	NR
Watt[a], 2017 [31]	4	PMP MO	Centrifugal	NR	NR	NR	98	NR	NR	46	NR	NR	NR
Cies[a], 2020 [32]	24	PMP MO	Centrifugal	NR	NR	NR	NR	NR	NR	NR	NR	NR	33

Study	Duration of laboratory (hour)	Type of oxygenator	Type of pump	Percent remaining (%)									
				Ampicillin	Cefotaxime	Flucloxacillin	Fluconazole	Gentamicin	Meropenem	Micafungin	Piperacillin	Vancomycin	Voriconazole
Raffaeli[a], 2020 [27]	24	PMP MO	Diagonal	NR	NR	NR	NR	NR	NR	NR	NR	62	20
Zhang[b], 2021 [28]	24	Silicone MO	Centrifugal	NR	NR	NR	NR	NR	45	67	NR	NR	60
Honeycutt[b], 2023 [29]	7	PMP MO	Centrifugal	NR	NR	NR	NR	NR	60	NR	NR	NR	NR
Lyster[b], 2023 [30]	24	PMP MO	Centrifugal	NR	NR	NR	NR	NR	NR	NR	NR	NR	73

PMP, polymethylpentane; MO, membrane oxygenator; NR, not reported; [a] pediatric circuitry; [b] adult circuitry.
